# A Sensitive, Rapid, On-Site Detection of Diflubenzuron in Food via a Colloidal Gold-Based Test Strip

**DOI:** 10.3390/foods15060977

**Published:** 2026-03-10

**Authors:** Yanni Zhu, Dan Wang, Wenqin Wu, Yinghua Deng, Zhaowei Zhang, Zhi-Quan Tian

**Affiliations:** 1 College of Chemistry and Molecular Sciences, Wuhan University, Wuhan 430072, China; 2023202030036@whu.edu.cn (Y.Z.); d202580180@hust.edu.cn (D.W.); 2 School of Bioengineering and Health, Hubei Hongshan Laboratory, Wuhan Textile University, Wuhan 430020, China; wenqin_wu@163.com; 3 State Key Laboratory of New Textile Materials and Advanced Processing Technologies, Wuhan Textile University, Wuhan 430020, China; 4 Hubei Key Laboratory of Purification and Application of Plant Anti-Cancer Active Ingredients, School of Chemistry and Life Science, Hubei University of Education, Wuhan 430205, China

**Keywords:** insecticide residue, point-of-care testing, visual screening, immunoassay, food safety

## Abstract

Diflubenzuron (DFB), a benzoylurea insecticide widely used in fruits, vegetables, cereals, and edible fungi, is increasingly detected in food. It has been linked to endocrine disruption, hematological effects, developmental toxicity, DNA damage, and ecological risks in aquatic organisms. These concerns, together with strict maximum residue limits, highlight the need for rapid, field-deployable detection methods. Herein, we developed a quantitative colloidal gold lateral-flow immunoassay for rapid DFB detection within 10 min. The optimized assay achieved a limit of detection (LOD) of 0.02 ng mL^−1^, a limit of quantification (LOQ) of 0.067 ng mL^−1^, and a linear range of 0.07–100 ng mL^−1^ (R^2^ = 0.9998), with high selectivity. Validation in eight food matrices (milk, chicken, mushrooms, pear, Chinese cabbage, rice, dried chili, and peanut) showed recoveries of 97.6–110.0% with RSDs of 2.1–4.9%. Results were consistent with LC-MS analysis, demonstrating that this assay provides a sensitive, practical, and rapid tool for screening DFB residues.

## 1. Introduction

The extensive application of pesticides in agriculture has substantially improved global crop production and mitigated the pressure on food supplies brought about by population growth [1]. However, the continued use of pesticides may lead to transboundary environmental migration, ecological risk accumulation, and increased residue levels in food [2,3]. Food monitoring data show that the mixed residues of multiple pesticides in fruits and vegetables are becoming increasingly common, with dietary exposure presenting complex cumulative or interactive effects, thereby increasing the potential for both acute and chronic health risks [4,5,6]. Diflubenzuron (DFB), a typical benzoylurea insecticide, is widely used on fruits, vegetables, and tea crops to control lepidopteran and coleopteran pests [7]. DFB has low acute toxicity to mammals. However, prolonged dietary exposure may cause endocrine disruption, hematological effects, and developmental toxicity. Long-term exposure to DFB in aquatic environments has been shown to significantly affect aquatic organisms such as fish, especially inducing oxidative stress and reproductive dysfunction [8,9,10]. Recent ecotoxicological studies have further demonstrated severe embryotoxic and developmental effects of DFB in fish models [9], while updated dietary risk assessments of pesticide residues in fresh vegetables and agricultural products continuously emphasize the critical need for vigilant food safety monitoring [11,12,13]. Moreover, toxicological studies in mammals have reported that diflubenzuron exposure can affect reproductive function and induce DNA damage in rodent and livestock models, suggesting that its metabolites may pose potential health risks to humans as well [14,15]. Therefore, despite DFB’s low acute toxicity, the ecological risks associated with long-term exposure, especially its impacts on ecosystem health and biodiversity, remain a major concern. China, the European Union, and the Codex Alimentarius Commission (CAC) have established strict maximum residue limits (MRLs) for DFB, with notable variations across different food categories. The MRLs for common foods such as fruits, vegetables, and grains are typically in the range of 0.1 to 2 mg kg^−1^ (FAO/WHO, 2023 [16]; EU, 2005 [17]; GB 2763-2021) [18].

Moreover, various techniques are employed for the detection of diflubenzuron. Techniques such as liquid chromatography–tandem mass spectrometry (LC-MS/MS), gas chromatography–mass spectrometry (GC-MS/MS) for heat-resistant matrices, and ultra-performance liquid chromatography–tandem mass spectrometry (UPLC-MS/MS) combined with QuEChERS pretreatment are widely adopted for the trace-level determination of target analytes with high sensitivity and quantitative accuracy [19,20,21]. However, these methods generally require expensive instrumentation, complex sample preparation, and specialized personnel. They are also time-consuming, which renders them unsuitable for rapid on-site screening [20,22]. Immunoassays, including enzyme-linked immunosorbent assays (ELISA), have attracted considerable attention due to their operational simplicity, short analysis time, and low cost. ELISA has also been applied for rapid field screening of diflubenzuron in cereal samples, demonstrating good analytical sensitivity with detection limits as low as 0.75 mg kg^−1^ [23]. However, traditional ELISA still involves lengthy incubation steps (typically 1–2 h) and relatively complex manual procedures, which limit its suitability for point-of-care applications [24]. Consequently, there is an increasing demand for cost-effective and user-friendly tools for on-site pesticide residue analysis. Lateral flow immunoassay (LFIA) test strips, which are simple to operate, respond rapidly, and require minimal resources, have been extensively applied for rapid field screening of pesticides [25,26,27,28]. Particularly in recent years, continuous optimizations in competitive LFIA configurations have further solidified their reliability, making LFIAs an indispensable and mainstream analytical tool for rapid detection and routine food safety inspections [29,30].

In this study, we developed a quantitative lateral-flow system utilizing gold nanoparticles to facilitate rapid field analysis of DFB in edible samples. By integrating a portable reader, a sensitive and quantitative detection mode was achieved. The optimization of colloidal gold–antibody conjugation and buffer composition contributes to the improved analytical performance of the assay. The method was systematically validated in terms of sensitivity, specificity, recovery, repeatability, and stability using authentic food samples.

While most previously reported LFIA approaches for DFB have primarily focused on qualitative or semi-quantitative screening, the present work demonstrates an effective strategy that combines a highly specific monoclonal antibody with a portable reader to enable both rapid visual inspection and reliable on-site quantification. Moreover, the validation of this quantitative platform across eight diverse and complex food matrices, ranging from dairy and meat products to fresh produce, highlights its broad applicability and practical potential for food safety monitoring, enabling reliable on-site quantitative analysis across diverse real-world samples.

## 2. Materials and Methods

### 2.1. Materials and Instruments

Monoclonal antibodies specific to diflubenzuron (DFB Abs) and the diflubenzuron–bovine serum albumin conjugate (DFB-BSA) were supplied by Shenzhen Kejie Industrial Development Co., Ltd. (Shenzhen, China). Goat anti-mouse IgG was obtained from Wuhan Boster Biological Technology Co., Ltd. (Wuhan, China). Diflubenzuron standard, bovine serum albumin (BSA), Tween-20, MES, polyvinylpyrrolidone K30 (PVP K30), sucrose, disodium ethylenediaminetetraacetate dihydrate (Na_2_EDTA·2H_2_O), and other analytical-grade reagents were purchased from Sinopharm Chemical Reagent Co., Ltd. (Shanghai, China). Assembly of the LFIA strips was performed using an XYZ3050 automated dispenser and an LM4000 laminator (BioDot Inc., Irvine, CA, USA). The completed sheets were then cut into individual strips with a CM4000 guillotine cutter (BioDot Inc., Irvine, CA, USA). The fluorescence signals of the strips were read and analyzed using a fluorescence immunoassay analyzer (CHF100) equipped with FSTools software (version 2.0.2, Guangzhou Tronho Medical Technology Co., Ltd., Guangzhou, China).

### 2.2. Synthesis and Characterization of Colloidal Gold

The synthesis of gold nanoparticles (AuNPs) was carried out using a modified trisodium citrate–mediated reduction protocol [31,32]. The brief procedure is as follows: Heat 100 mL of a dilute HAuCl_4_ solution (0.01%, *w*/*v*) in a three-neck flask until it reaches boiling. While maintaining continuous stirring, quickly introduce 1.0 mL of a 1% (*w*/*v*) trisodium citrate aqueous solution. The reaction mixture was allowed to continue boiling for approximately 10 min. It was then removed from the heat and cooled to room temperature. Store the resulting solution in the dark at 4 °C until required.

The UV-visible profiles of the gold nanoparticles were measured within the 450–600 nm range to track their surface plasmon resonance (SPR) peak, confirming the characteristic optical properties of the synthesized AuNPs [33]. The AuNP suspension was passed through a 0.22 μm filter before further experiments to eliminate residual particulates. The particle morphology and size distribution were characterized using transmission electron microscopy [34]. For TEM imaging, diluted nanoparticle dispersions were deposited onto carbon-coated copper grids and allowed to dry naturally in air [35]. The hydrodynamic size and ζ-potential of the nanoparticles were characterized in triplicate (n = 3) with a dynamic light scattering analyzer to assess their colloidal stability and surface charge properties [36]. Ultrapure water for all experimental procedures was supplied by a Milli-Q water purification unit. The resulting AuNPs were used for subsequent antibody conjugation [37].

### 2.3. Conjugation and Characterization of DFB Monoclonal Antibody with Colloidal Gold

The resulting AuNP dispersion was gently mixed at ambient temperature, and its acidity was gradually tuned to pH 7.5 with a 0.1 mol/L potassium carbonate solution to provide optimal conditions for antibody attachment [38]. An accurately prepared DFB monoclonal antibody solution (optimized at 5 μg mL^−1^) was introduced dropwise into every milliliter of the AuNP suspension, after which the mixture was gently agitated for 15 min to facilitate stable attachment of the antibody onto the nanoparticle surface through electrostatic and hydrophobic interactions [39]. Subsequently, 10% (*w*/*v*) BSA was introduced to passivate the remaining active sites, and the mixture was left to stand for another 15 min to complete the blocking process [40]. The resulting conjugates were separated by high-speed centrifugation (13,500× *g*, 20 min, 4 °C), after which the pellet was gently dispersed in a preservation buffer composed of 1% (*w*/*v*) PVP, 0.05% (*w*/*v*) NaN_3_, 5% (*w*/*v*) sucrose, and 10 mmol/L PBS (pH 7.4) [38]. The final AuNP-antibody combinations were stored at 4 °C prior to use to maintain their stability and immunological activity [41].

### 2.4. Preparation of DFB Colloidal Gold Immunochromatographic Test Strips

The fabrication of the DFB immunochromatographic device began with the preparation of individual membrane components. First, the test (T) and control (C) lines were dispensed onto a nitrocellulose (NC) membrane using a programmable membrane dispenser at a rate of 0.8 μL cm^−1^. The region corresponding to the T line was coated with the DFB-BSA conjugate (1.0 mg mL^−1^ in PBS, pH 7.4), while the C line region was coated with goat anti-mouse IgG (2.0 mg mL^−1^). The NC membrane was then dried at 37 °C for 2 h. Meanwhile, the conjugate pad was prepared by pre-treating a glass-fiber membrane with a PBS buffer (pH 7.4) containing 1% (*w*/*v*) polyvinylpyrrolidone (PVP), 0.05% (*w*/*v*) NaN_3_, and 5% (*w*/*v*) sucrose to enhance conjugate release and chromatographic stability. The pre-formed Au-mAb complex was then evenly applied to this treated pad and dried.

Following component preparation, the test strip was fabricated by sequentially assembling the sample pad, the AuNP-antibody conjugate pad, the treated NC membrane, and the wicking pad onto a plastic backing support. Each component overlapped the adjacent one by approximately 2 mm to ensure uninterrupted capillary-driven fluid migration. The fully assembled card was equilibrated for 12 h in a constant-temperature and humidity chamber. Finally, the assembly was cut into 3.5 mm-wide test strips using a slitting machine. The finished strips were sealed in aluminum foil pouches with desiccant and stored in the dark at 4 °C until use.

The resulting immunochromatographic assay operates on a competitive inhibition mechanism. When the sample solution migrates through the sample pad, free DFB competes with the immobilized DFB-BSA on the T line for binding to the colloidal gold-antibody conjugate, thereby modulating the color intensity of the T line. Simultaneously, the C line captures the excess unbound conjugate via the immobilized anti-mouse IgG, producing a constant reference signal to confirm the integrity of the assay system.

### 2.5. Optimization of Antibody Labeling Conditions on Colloidal Gold

A set of ten 2 mL centrifuge tubes, each containing 1 mL of colloidal gold solution and labeled 1–10, was then prepared. Different volumes of 0.1 mol/L K_2_CO_3_ were added to adjust the pH. After gentle mixing, 200 μL of anti-DFB monoclonal antibody solution (0.1 mg mL^−1^) was added to each tube. The suspensions were incubated at ambient temperature for 2 h to promote antibody binding to the AuNP surface. Following incubation, each tube was visually inspected for precipitation. Supernatants from tubes without visible precipitation were collected and analyzed using a UV-Vis spectrophotometer over the range of 450–600 nm. The morphology and position of the surface plasmon resonance (SPR) peaks were compared. The optimal pH was defined as the condition under which the SPR peak remained sharp, centered near 520 nm, and showed no significant red shift or decrease in absorption intensity.

To optimize the antibody dosage, another set of ten 2 mL centrifuge tubes was prepared, each containing 1 mL of colloidal gold dispersion adjusted to the optimal pH [42]. Volumes of 10, 20, 30, 40, 50, 60, 70, 80, 90, and 100 μL of anti-DFB monoclonal antibody solution (0.1 mg mL^−1^) were added, corresponding to final antibody concentrations of 1–10 μg mL^−1^. The mixtures were gently mixed and incubated at ambient temperature for 5 min. Subsequently, 100 μL of 10% (*w*/*v*) NaCl was added to each tube to assess colloidal stability, followed by incubation for an additional 5 min [43]. Color changes were visually observed, and absorbance spectra were recorded using a UV-Vis spectrophotometer. The optimal antibody concentration was defined as the lowest amount that maintained colloidal gold stability, characterized by a persistent red color without noticeable blue shift or precipitation and an SPR peak centered around 520 nm.

### 2.6. Evaluating the Performance of the Test Strip

DFB working dilutions (0, 0.05, 0.1, 1, 10, 25, 50, 75, 100, and 110 ng mL^−1^) were prepared by diluting the 1 mg mL^−1^ DFB stock with the sample extraction buffer. For each concentration, 100 μL standard analyte was dispensed onto the sample pad of the strip. After a 5 min standing period at ambient temperature, the strips were scanned with a colloidal gold reader (CHF100, Wondfo Biotech Co., Ltd., Guangzhou, China) to obtain the T- and C-line reflectance values. The instrument-derived data were exported and processed using a smartphone-based application to calculate the T/C ratio, plot the calibration curve, and perform linear regression. The adequacy of the simple unweighted linear regression over the established working range was confirmed by evaluating the residual distribution, which verified the assumption of homoscedasticity. LOD and LOQ were calculated using the formulas LOD = 3σ/k and LOQ = 10σ/k. Here, σ explicitly represents the standard deviation of the T/C ratios obtained from 20 blank measurements (n = 20), and k is the slope of the linear calibration curve. The smartphone was used solely for data handling, calculation, and visualization; no smartphone imaging was performed.

To evaluate specificity, structurally related compounds and common pesticides were tested as potential interferents. Three benzoylurea analogs (benzoylphenylurea (BPB), flubenzuron (FB), and chlorfluazuron (CFB)) were assessed for cross-reactivity at 100 ng mL^−1^, the same concentration as DFB. Seven other common pesticides (prophos (PF), dichlorvos (DDVP), chlorpyrifos (CPF), phosphamidon (PM), cypermethrin (CPM), malathion (MT), and iprodione (IPD)) were evaluated at concentrations 2–5 times higher than DFB (200–500 ng mL^−1^). DFB at 100 ng mL^−1^ served as the positive control. All experiments were conducted under consistent conditions, and the T/C ratios were calculated to determine relative responses. Since a single-concentration screening approach was utilized instead of full IC_50_ curve plotting (consistent with the rapid detection purpose of the proposed method), cross-reactivity was evaluated by calculating the relative signal difference compared to the negative control. Compounds showing ≤10% relative difference were considered non-interfering, indicating good specificity of the test strips.

To assess storage stability, three batches of strips were sealed in foil sachets containing desiccant and stored at 4 ± 2 °C for 60 days. Sampling was performed on days 0, 10, 20, 30, 40, 50, and 60. At each time point, 10 strips from each batch were tested using a DFB standard at the LOQ level. T/C values were measured as described above, and the mean and coefficient of variation (CV) were calculated. Stability was considered acceptable when CV values remained ≤10% throughout the storage period. The precision of the strips was assessed by examining intra-day precision (within-batch repeatability) and inter-day precision, as well as between-batch reproducibility, in accordance with the established validation criteria of the SANTE/11312/2021 guidelines [44]. For intra-day precision (within-batch repeatability), six strips from the same batch were used to test three concentrations of DFB (0.1, 10, and 100 ng mL^−1^) on the same day. T/C values, recovery, and CV were calculated, and a CV ≤ 10% indicated acceptable repeatability. For inter-day precision, the same three concentrations were tested in six replicates on three consecutive days. For between-batch reproducibility, six batches of test strips were tested in triplicate at the same concentrations. Mean T/C values and CVs across batches were calculated, with a CV ≤ 15% considered acceptable. All experiments were conducted in triplicate, with data presented as mean ± SD.

### 2.7. Sample Pretreatment

Sample preparation was performed using a modified QuEChERS procedure to ensure that the detection limits met the Maximum Residue Limits (MRLs) specified in the National Food Safety Standard of China (GB 2763–2021 [18]). All 8 representative food samples (milk, chicken, fresh mushrooms, pear, Chinese cabbage, rice, dried chili peppers, and peanuts) were processed using the same preprocessing method. A 5.00 g portion of each homogenized sample (±0.01 g) was weighed into a 50 mL centrifuge tube, followed by the addition of 10 mL of deionized water. The mixture was vortex-mixed for 1 min and allowed to stand for 30 min to ensure complete rehydration. Subsequently, 10 mL of acetonitrile was added, and the tube was shaken vigorously for 20 min to extract DFB. Phase separation was induced by adding 2.5 g of sodium chloride, followed by vortex mixing for 30 s. For samples with clear phase separation (including milk, pear, rice, and fresh mushrooms), the upper acetonitrile phase was directly collected. For samples with unclear phase separation (including chicken, peanuts, dried chili peppers, and Chinese cabbage), centrifugation at 2500× *g* for 5 min was performed first to achieve complete phase separation. The upper acetonitrile phase was then collected, filtered through a 0.22 μm organic-phase membrane, and adjusted to a final volume of 10.0 mL with acetonitrile for quantitative analysis. The extract was stored at 4 °C until further use. Prior to LFIA analysis, the extract was diluted five-fold with PBS (0.01 mol/L, pH 7.4). This procedure provided sufficient matrix cleanup for immunochromatographic detection, including samples with relatively high lipid or protein contents. As a result, the overall sample preparation introduced a total dilution factor of 10, consisting of a two-fold dilution during extraction (5.00 g sample into 10.0 mL solvent) followed by a five-fold buffer dilution. Accordingly, the limit of detection in food matrices (0.2 μg kg^−1^) was calculated by multiplying the detection limit of the test strip in buffer (0.02 ng mL^−1^) by this dilution factor.

## 3. Results

### 3.1. Detection Principle

**Figure 1:**
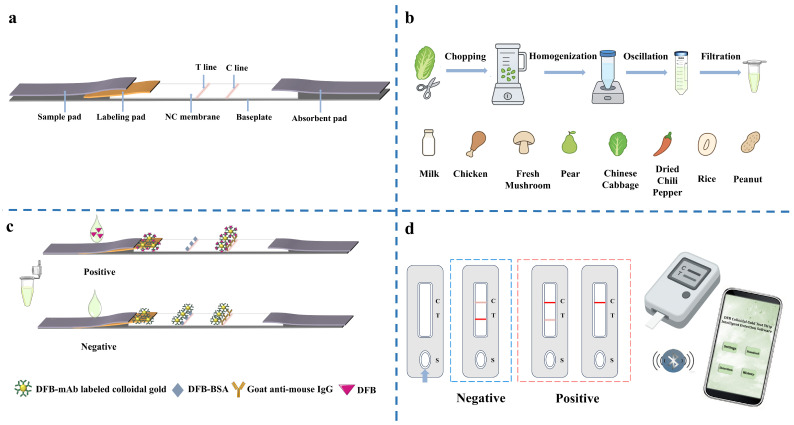
Schematic illustration of DFB detection using a colloidal gold immunochromatographic strip (C: Control line, T: Test line, S: Sample well). (**a**) Schematic structure of the DFB colloidal gold strip; (**b**) Sample matrices and pretreatment for DFB detection; (**c**) Principle of DFB immunoassay (Positive/Negative); (**d**) Smartphone-assisted detection and quantitative analysis.

The quantitative detection of DFB is performed using a gold nanoparticle (AuNP) immunochromatographic strip that exploits specific antigen–antibody interactions (Figure 1). The nitrocellulose membrane is functionalized with a test line (T line) immobilized with DFB–bovine serum albumin (DFB-BSA) conjugate to capture AuNP-labeled anti-DFB monoclonal antibodies, and a control line (C line) modified with a secondary antibody captures excess unbound AuNP-antibody conjugates to verify strip integrity. A portable strip reader (CHF100, Wondfo Biotech Co., Ltd., Guangzhou, China; detection wavelength 520–550 nm) measures the transmitted light intensity at the T and C line regions (denoted IT and IC, respectively). Darker T-line bands absorb more light, resulting in lower IT values, whereas the C line maintains a stable IC across different DFB concentrations. The ratio T/C = IT/IC positively correlates with DFB concentration. For samples containing DFB, free DFB binds to AuNP-antibody conjugates, reducing their availability to interact with the T line and thus lightening its color (higher IT). For DFB-free samples, most AuNP-antibody conjugates bind the T line, producing a dark T line (low IT) while the C line remains stable, yielding a low T/C ratio. DFB concentrations in unknown samples are quantified by correlating measured T/C ratios with a pre-established calibration curve. Data processing, including T/C calculation, linear regression, and visualization, is performed via a customized smartphone application. The entire detection process is completed in approximately 5 min, enabling rapid on-site quantitative analysis.

### 3.2. Characterization of Colloidal Gold

**Figure 2:**
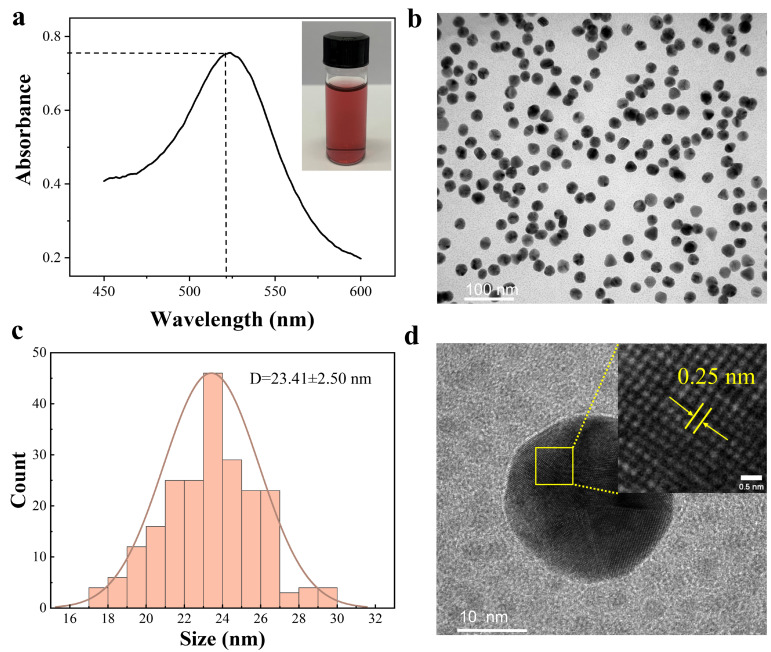
Characterization of colloidal gold nanoparticles (AuNPs). (**a**) Photograph of the colloidal gold solution and its UV–Vis absorption spectrum; (**b**) Low-magnification TEM image of AuNPs; (**c**) Particle size distribution of AuNPs, showing an average diameter of 23.41 ± 2.50 nm; (**d**) High-resolution TEM image of a single AuNP displaying lattice fringes with an interplanar spacing of 0.25 nm, corresponding to the (111) plane of face-centered cubic gold.

Colloidal gold nanoparticles were synthesized via the trisodium citrate reduction method (Figure 2a). The obtained colloidal solution exhibited a uniform wine-red color without visible precipitation or impurities, indicating good colloidal stability. UV-Vis spectroscopy displayed a characteristic surface plasmon resonance absorption peak at 523 nm, confirming the successful formation of AuNPs. TEM analysis revealed that the synthesized AuNPs were predominantly spherical with good dispersibility (Figure 2b). Statistical analysis showed an average particle diameter of 23.41 ± 2.50 nm (Figure 2c). High-resolution TEM further resolved well-defined lattice fringes with an interplanar spacing of 0.25 nm, which can be indexed to the (111) crystal plane of face-centered cubic gold (Figure 2d), demonstrating the crystalline nature of the synthesized AuNPs. The hydrodynamic diameter was further measured by DLS (n = 3) to be 26.8 ± 1.2 nm.

### 3.3. Optimization of pH and DFB Monoclonal Antibody (DFB Abs) Concentration

To optimize the colloidal stability, different volumes of 0.1 mol/L K_2_CO_3_ were added to adjust the pH of the gold dispersion. After conjugation with diflubenzuron (DFB) monoclonal antibody, the stability of the colloidal gold system was evaluated using a 10% (*w*/*v*) NaCl-induced aggregation assay. The results showed that the gold dispersion maintained a stable wine-red appearance over a pH range of 7.0–8.5 (Figure S1c). Ultraviolet-visible spectrophotometric analysis further demonstrated that the colloidal gold solution exhibited maximum absorbance at 529 nm at pH 7.5 (Figure S1a).

Subsequently, after adjusting the pH to 7.5, AuNPs were labeled with different concentrations of DFB monoclonal antibody and assessed by the same NaCl-induced aggregation assay. The results indicated that the colloidal gold solution retained a stable wine-red color at antibody concentrations of 5 μg mL^−1^ and above. Based on these findings, 5 μg mL^−1^ was selected as the optimal antibody labeling concentration. UV-Vis spectra confirmed that the gold dispersion labeled with 5 μg mL^−1^ DFB monoclonal antibody exhibited a characteristic absorption peak at 529 nm (Figure S1b,d).

### 3.4. Characterization of Antibody-Modified Colloidal Gold Probes

**Figure 3:**
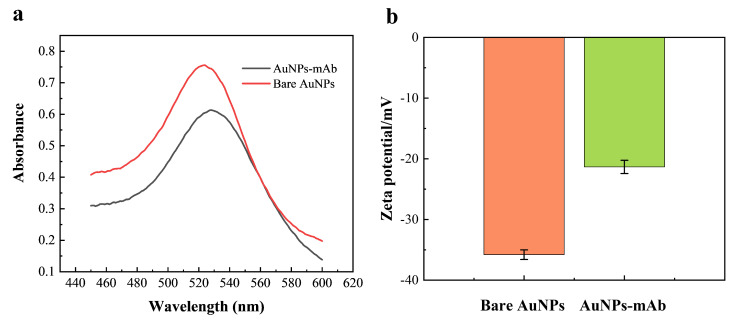
Characterization of colloidal gold before and after conjugation with anti-diflubenzuron antibody. (**a**) UV-Vis spectra of colloidal gold and colloidal gold-labeled antibody; (**b**) Zeta potential of colloidal gold and colloidal gold-labeled antibody.

Under optimized conditions, a colloidal gold probe conjugated with DFB antibodies was prepared and characterized by UV-visible spectroscopy (Figure 3). Upon conjugation with DFB antibodies, followed by BSA blocking, the maximum absorbance peak shifted from 523 nm to 529 nm, reflecting an increased local refractive index and hydrodynamic diameter. This observation was further supported by DLS analysis, which revealed an increase in hydrodynamic size, consistent with protein adsorption on the AuNP surface and confirming successful antibody attachment. The zeta potential changed from −35.8 mV to −21.3 mV (n = 3), indicating an altered surface charge while maintaining sufficient colloidal stability (|ζ| > 20 mV). In addition, the dispersion color changed from a bright red to a deeper red, providing visual evidence of the formation of a stable colloidal gold probe suitable for subsequent immunoassays.

### 3.5. Optimization of Test Strip Conditions

**Figure 4:**
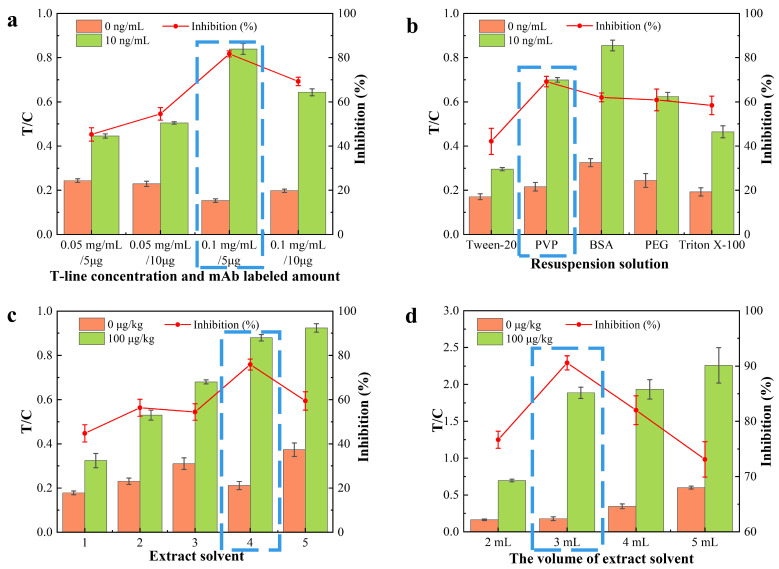
Optimization of DFB colloidal gold test strips and sample pretreatment (T/C values and inhibition rates, the blue boxes indicate the optimal parameters). (**a**) T-line concentration (mg mL^−1^) and amount of labeled mAb; (**b**) Resuspension solution type; (**c**) Extraction solvent type; (**d**) Volume of extraction solvent (mL).

The optimization results are presented in Figure 3. An optimal antibody concentration of 5 μg mL^−1^ was utilized, and specific surfactants were incorporated to facilitate biomolecular recognition. Resuspending the AuNP-antibody conjugates in a 20 mmol/L PBS buffer (pH 7.4) containing 5% (*w*/*v*) PVP significantly improved both the inhibition efficiency and the T/C ratio. This resuspension medium was critical for maintaining the stability of the probe, as confirmed by the consistent hydrodynamic diameter and zeta potential. Furthermore, the sample extraction and treatment protocol was optimized to suppress matrix interference. As detailed in Section 2.7, a modified QuEChERS procedure was employed, involving the extraction of 5.00 g of homogenized food sample with 10 mL of deionized water and 10 mL of acetonitrile. This approach effectively neutralized the influence of complex food components such as carbohydrates, pigments, and secondary metabolites [25], preserving both the sensitivity and accuracy of the test strips (Figure 4a–d). The systematic optimization of the buffer composition and extraction ratio ensured that the assay could reliably meet the required analytical performance standards across various food matrices [26].

### 3.6. Detection Performance of the Test Strip

**Figure 5:**
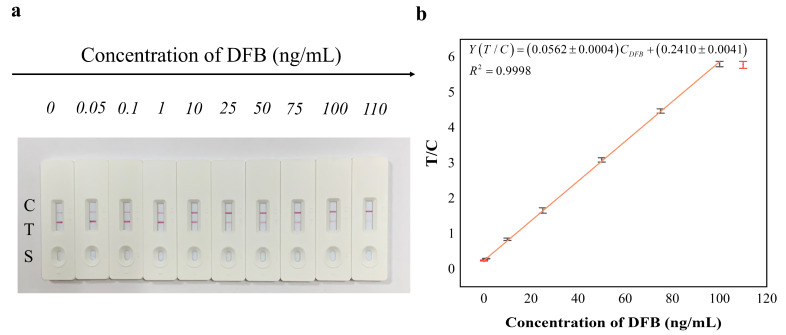
Quantitative detection and calibration curve of DFB by colloidal gold immunochromatographic strip (C: Control line, T: Test line, S: Sample well). (**a**) Visual detection of DFB at concentrations (ng mL^−1^): 0, 0.05, 0.1, 1, 10, 25, 50, 75, 100, 110 (corresponding to lanes 1–10); (**b**) Calibration curve of DFB with linear relationship (R^2^ = 0.9998).

The T/C ratios of the standard solutions were plotted against their respective DFB concentrations to construct a calibration curve [31]. The resulting curve was described by the equation Y (T/C) = (0.0562 ± 0.0004) × C_DFB_ + (0.2410 ± 0.0041) (Figure 5). The adequacy of the unweighted linear regression model across the wide dynamic range was confirmed by residual analysis, which demonstrated homoscedasticity with randomly distributed residuals. Under the optimized conditions, the limit of detection (LOD, calculated as 3σ/k) and the limit of quantification (LOQ, calculated as 10σ/k) were determined to be 0.02 ng mL^−1^ and 0.067 ng mL^−1^, respectively, where σ represents the standard deviation of the T/C ratio from twenty independent blank measurements and k is the slope of the calibration curve. The assay exhibited a linear response over the range of 0.07 ng mL^−1^–100 ng mL^−1^, with an excellent coefficient of determination (R^2^ = 0.9998). A threshold of 100 ng mL^−1^ was set; DFB concentrations ≥ 100 ng mL^−1^ were clearly identified as positive, whereas concentrations below 0.07 ng mL^−1^ could not be distinguished from negative samples. To quantitatively evaluate the matrix effect (ME), calibration curves were constructed using both solvent-based standards and matrix-matched standards for all eight food matrices. The ME was calculated as: ME (%) = (Slope matrix/Slope solvent − 1) × 100% The results showed that the ME values for all matrices ranged from −8.4% to 11.2%, which falls well within the negligible range of ±20%. These findings demonstrate that the optimized sample preparation (10-fold total dilution) and the incorporation of specific surfactants in the resuspension buffer (as detailed in Section 3.5) effectively eliminated matrix interference. Furthermore, the intra-day precision, inter-day precision, and batch-to-batch reproducibility of the assay were evaluated at three DFB concentration levels (0.1, 10, and 100 ng mL^−1^). As summarized in Table S1, the relative standard deviations (RSDs) ranged from 5.5% to 10.3%, demonstrating good assay precision and high reproducibility of the manufactured test strips.

Compared with conventional DFB detection methods lacking on-site capability (Table 1), the newly developed AuNP-based immunochromatographic strip exhibits a lower LOD and a broader linear range. Notably, the proposed method not only achieves superior sensitivity (0.02 ng mL^−1^) but also demonstrates validated reliability across a wider range of food categories (n = 8) compared with the specialized methods listed in Table 1. While classical instrumental analyses offer excellent accuracy, they are often limited by lengthy sample preparation procedures and high operational costs [45,46]. Traditional ELISA methods, although sensitive, typically require prolonged incubation steps and laboratory-based operation [23]. As comprehensively compared in Table 1, the proposed LFIA effectively addresses these limitations by enabling rapid, real-time, on-site quantitative analysis. In addition, it provides advantages in sensitivity, portability, and visual readout over previously reported techniques. This performance is consistent with current trends in agricultural and food safety monitoring, where nanoparticle-amplified lateral flow assays are increasingly recognized as practical alternatives to laboratory-based methods due to their rapid response, portability, and cost-effectiveness [29,47,48,49].

**Figure 6:**
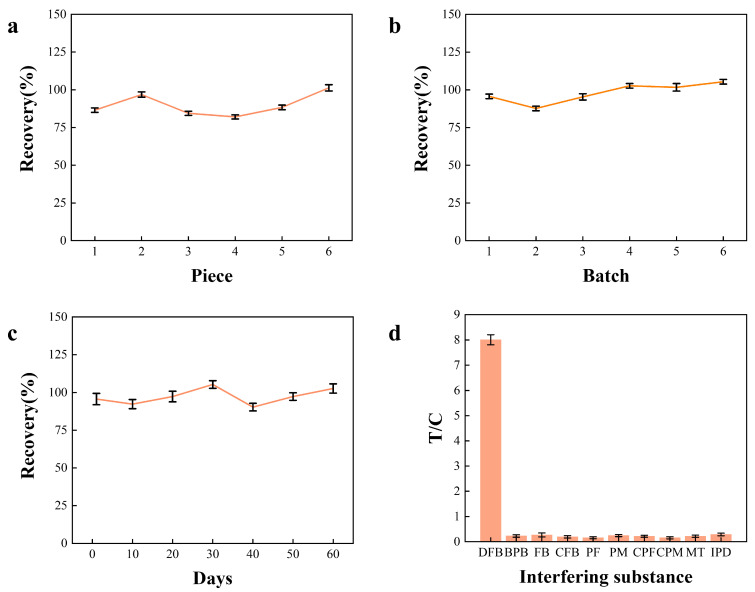
Performance evaluation of the DFB colloidal gold immunochromatographic strip. (**a**) Intra-day precision (within-batch repeatability). (**b**) Between-batch reproducibility. For (**a**,**b**), results are presented at a representative spiked DFB concentration of 10 ng mL^−1^; full precision validation data, including inter-day precision, are provided in Table S1. (**c**) Storage stability expressed as recovery over 60 days at 4 ± 2 °C. (**d**) Specificity evaluation showing T/C ratios for DFB and potential interfering substances (BPB, FB, CFB, PF, PM, CPF, CPM, MT, and IPD).

The specificity of the test strips was evaluated using several structurally related compounds, including BPB, FB, CFB, PF, PM, CPF, CPM, MT, and IPD (Figure 6d). Three benzoylurea analogs (BPB, FB, and CFB) were assessed at 100 ng mL^−1^, while seven other common pesticides (PF, PM, CPF, CPM, MT, and IPD) were evaluated at higher concentrations (200–500 ng mL^−1^). No apparent inhibition curves were observed for these structural analogs under the tested conditions, and therefore an exact cross-reactivity calculation based on half-maximal inhibitory concentrations (IC_50_) was not applicable. Instead, specificity was assessed via relative signal differences, with all interferents exhibiting a variation of ≤10% compared to the blank control, indicating negligible cross-reactivity and confirming the high selectivity of the strip toward DFB.

To evaluate the performance of the developed test strips, recoveries and precision were assessed across eight food matrices. The results showed that recoveries ranged from 97.6% to 110.0%, with RSDs of 2.1–4.9%, demonstrating excellent accuracy and reproducibility. The detailed results for intra-day and inter-day precision, as well as batch-to-batch reproducibility, are summarized in Table S1. These analytical performance metrics fully comply with the criteria established by recognized international and national validation guidelines, including the European Union SANTE guidelines (SANTE/11312/2021) [44], the Codex Alimentarius Guidelines (CAC/GL 90-2017) [52], and the Chinese agricultural industry standard (NY/T 4048-2021) [53]. According to these standards, the acceptable recovery range for pesticide residue analysis at these concentration levels is typically 60–120%, with an RSD threshold of ≤0% or ≤25%. Specifically, the spiking levels (expressed in µg kg^−1^ within the original matrices) and the corresponding recoveries for milk were 98.3–101.9% (RSD 2.5–4.0%), chicken 97.6–101.0% (RSD 2.9–4.5%), fresh mushrooms 99.6–103.8% (RSD 2.2–4.6%), pear 97.8–103.4% (RSD 3.3–4.9%), Chinese cabbage 101.8–105.3% (RSD 2.1–4.0%), rice 98.0–101.6% (RSD 2.7–4.4%), dried chili 101.2–110.0% (RSD 3.1–4.4%), and peanut 98.0–102.9% (RSD 3.9–4.7%) (Figure 6c). These results indicate that the developed strips can reliably detect DFB residues in various food matrices, providing a rapid and practical alternative to LC-MS analysis. To formally evaluate the analytical consistency between the developed LFIA and the standard liquid chromatography–mass spectrometry (LC–MS) method, a linear regression comparison was performed using the detected concentrations across all 24 spiked food samples (Table 2). As shown in Figure S2, the regression analysis yielded the equation Y = 0.99X + 0.37 with a high coefficient of determination (R^2^ = 0.99). The slope and intercept were within the 95% confidence intervals of [0.96, 1.02] and [−0.15, 0.89], respectively, indicating no significant constant or proportional bias. Furthermore, a paired *t*-test was conducted on the data sets from both methods, yielding a calculated t-value of 1.41 and a *p*-value of 0.17 (*p* > 0.05, n = 24). These statistical results demonstrate that the developed LFIA method provides results equivalent to the standard LC–MS method, confirming its practical reliability and accuracy for the on-site detection of DFB residues.

### 3.7. Limitations of the Proposed LFIA Method

Despite the advantages of rapid response, portability, and on-site applicability, the proposed LFIA also has certain limitations. First, as an antibody-based assay, its analytical performance is inherently dependent on antibody specificity. Although negligible cross-reactivity (CR < 0.1%) was observed for the tested analogues in this study, potential interference from untested structurally related compounds or emerging metabolites cannot be completely excluded. Second, while the developed LFIA enables quantitative analysis, its analytical precision (RSDs of 5.5–10.3%) remains lower than that of laboratory-based instrumental techniques such as LC–MS/MS, which are still required for confirmatory analysis and identifying unknown contaminants. Finally, although the method demonstrates good applicability across multiple food matrices, complex matrices with exceptionally high fat or pigment contents (e.g., certain spices or highly processed oils) may require additional dilution or specialized cleanup steps to further minimize matrix effects beyond the standard 10-fold dilution. Overall, the proposed LFIA is primarily intended as a rapid screening and on-site quantitative tool, complementing rather than replacing conventional instrumental methods in food safety monitoring.

## 4. Conclusions

A highly sensitive gold nanoparticle (AuNP)-based immunochromatographic strip was developed for rapid on-site detection of DFB residues in food samples. By optimizing nanoparticle synthesis, antibody conjugation, and strip assembly, a stable and reproducible quantitative system was established, enabling reliable measurements within 10 min using a portable strip reader. The optimized assay achieved a detection limit of 0.02 ng mL^−1^ in the working buffer, corresponding to a matrix-based limit of detection of 0.2 ng g^−1^ after accounting for sample pretreatment dilution factors. The assay exhibited a quantification limit of 0.067 ng mL^−1^ and a broad linear response range of 0.07–100 ng mL^−1^ in buffer, equivalent to 0.7–1000 ng g^−1^ in food matrices, with an excellent correlation coefficient (R^2^ = 0.9998). The test strips demonstrated good repeatability and inter-batch reproducibility (CV < 10%), satisfactory recovery rates (97.6–110.0%), and good storage stability for at least 60 days at 4 °C. In addition, excellent agreement with standard LC–MS analysis further confirmed the accuracy and practical reliability of the proposed method. Overall, this approach, integrating a rapid colloidal gold immunoassay with a portable quantitative readout, provides a sensitive, practical, and economical solution for routine screening of DFB residues in diverse food matrices. The key novelty of this research lies in translating a conventional dip-and-read lateral flow strip into a robust, reader-assisted quantitative platform, thereby improving analytical reliability while maintaining rapid on-site applicability. This enhanced versatility and reliability highlight its value as a practical tool for routine multi-matrix food safety monitoring.

## Figures and Tables

**Table 1:** Comparison of the proposed LFIA with other reported methods for DFB detection.

Method	LOD ^g^ (µg kg^−1^)	Linear Range (µg kg^−1^)	Detection Time (min)	Portability	Visualization	Ref.
LC-MS/MS ^a^	0.01	0.01–0.2	>15	No	No	[45]
UPLC-MS/MS ^b^ (QuEChERS) ^c^	0.10–1.5	1–5000	15	No	No	[46]
HPLC-luminol chemiluminescence ^d^	2.5	50–500	>15	No	No	[50]
MIP-PEC sensor ^e^	0.00125	0.01–1000	>15	No	No	[51]
Rapid immunoassay (ELISA) ^f^	750	750–25,000	15	Yes	Yes	[23]
This work	0.2	0.7–1000	10	Yes	Yes	-

^a^ LC-MS/MS: Liquid Chromatography-Tandem Mass Spectrometry. ^b^ UPLC-MS/MS: Ultra Performance Liquid Chromatography-Tandem Mass Spectrometry. ^c^ QuEChERS: Quick, Easy, Cheap, Effective, Rugged, and Safe sample pretreatment method. ^d^ HPLC: High Performance Liquid Chromatography. ^e^ MIP-PEC sensor: Molecularly Imprinted Polymer-Photoelectrochemical sensor. ^f^ ELISA: Enzyme-Linked Immunosorbent Assay. ^g^ LOD: Limit of Detection. Note: For this work, the LOD and linear range were initially determined in the working buffer solution and are expressed as ng mL^−1^. Considering the overall dilution factor of 10 applied during sample preparation (5 g sample extracted into 10 mL solvent, followed by a 5-fold dilution prior to LFIA analysis), the corresponding matrix-based performance translates to an LOD of 0.2 μg kg^−1^ and a linear range of 0.7–1000 μg kg^−1^ in food samples.

**Table 2:** Validation of the proposed LFIA method by comparison with LC-MS in actual food samples (n = 3).

		This Work			LC-MS		
Samples	Spiked (µg kg^−1^)	Detected (µg kg^−1^)	Recovery (%)	RSD (%)	Detected (µg kg^−1^)	Recovery (%)	RSD (%)
Milk	0.0	0.04 ± 0.001	-	2.5	0.04 ± 0.001	-	2.3
	50.0	51.0 ± 1.40	101.9	2.7	52.0 ± 1.60	103.9	3.1
	500.0	506.0 ± 20.10	101.2	4.0	508.0 ± 19.80	101.5	3.9
	1000.0	983.0 ± 24.30	98.3	2.5	985.0 ± 23.50	98.5	2.4
Chicken	0.0	0.07± 0.002	-	2.9	0.07 ± 0.002	-	2.7
	50.0	49.0 ± 1.90	97.9	3.9	50.0 ± 2.00	99.9	4.0
	500.0	505.0 ± 22.50	101.0	4.5	507.0 ± 21.80	101.2	4.3
	1000.0	976.0 ± 41.60	97.6	4.3	978.0 ± 40.20	97.8	4.1
Fresh mushroom	0.0	0.09 ± 0.002	-	2.2	0.09 ± 0.002	-	2.0
	50.0	52.0 ± 1.80	103.8	3.5	53.0 ± 1.90	105.8	3.6
	500.0	513.0 ± 20.20	102.6	4.6	515.0 ± 19.50	103.0	3.8
	1000.0	996.0 ± 26.50	99.6	2.7	998.0 ± 25.80	99.8	2.6
Pear	0.0	0.16 ± 0.006	-	3.8	0.16 ± 0.005	-	3.1
	50.0	51.0 ± 1.70	101.7	3.3	52.0 ± 1.80	103.7	3.5
	500.0	489.0 ± 23.60	97.8	4.8	491.0 ± 22.80	98.0	4.6
	1000.0	1034.0 ± 50.40	103.4	4.9	1036.0 ± 49.20	103.6	4.7
Chinese cabbage	0.0	0.19 ± 0.004	-	2.1	0.19 ± 0.003	-	1.8
	50.0	52.0 ± 1.50	103.6	2.9	53.0 ± 1.60	105.6	3.0
	500.0	509.0 ± 20.20	101.8	4.0	511.0 ± 19.60	102.2	3.8
	1000.0	1053.0 ± 40.90	105.3	3.9	1055.0 ± 39.80	105.5	3.8
Rice	0.0	ND ^a^	-	-	ND ^a^	-	-
	50.0	49.0 ± 1.30	98.0	2.7	49.8 ± 1.40	99.6	2.8
	500.0	508.0 ± 22.30	101.6	4.4	510.0 ± 21.50	102.0	4.2
	1000.0	987.0 ± 42.50	98.7	4.3	989.0 ± 41.20	98.9	4.2
Dried chili pepper	0.0	5.9 ± 0.20	-	3.4	5.9 ± 0.20	-	3.2
	50.0	55.0 ± 1.70	110.0	3.1	56.0 ± 1.80	112.0	3.2
	500.0	512.0 ± 22.60	101.2	4.4	514.0 ± 21.90	101.4	4.3
	1000.0	1048.0 ± 36.50	104.8	3.5	1050.0 ± 35.80	105.0	3.4
Peanut	0.0	ND ^a^	-	-	ND ^a^	-	-
	50.0	49.0 ± 2.20	98.0	4.5	50.0 ± 2.30	100.0	4.6
	500.0	509.0 ± 23.90	101.8	4.7	511.0± 23.20	102.2	4.5
	1000.0	1029.0 ± 40.50	102.9	3.9	1031.0± 39.80	103.1	3.8

^a^ ND: Not Detected. Note: For consistency across both solid and liquid matrices, the concentrations for milk samples are expressed as mass-based equivalents assuming a density of approximately 1 kg L^−1^ (1 μg L^−1^ ≈ 1 μg kg^−1^).

## Data Availability

The original contributions presented in this study are included in the article/Supplementary Materials. Further inquiries can be directed to the corresponding authors.

## Supplementary Materials

The following supporting information can be downloaded at: https://www.mdpi.com/article/10.3390/foods15060977/s1, Figure S1. Optimization of pH and antibody concentration for colloidal gold immunoassay. (a) UV-Vis spectra of colloidal gold at different pH values; (b) UV-Vis spectra of colloidal gold with varying anti-diflubenzuron antibody concentrations; (c) Photographs of colloidal gold solutions at different pH values; (d) Photographs of colloidal gold solutions with varying anti-diflubenzuron antibody concentrations. Figure S2. Correlation between the developed LFIA and the reference LC–MS method for DFB detection in spiked food samples. Table S1. Intra-day precision, inter-day precision, and batch-to-batch reproducibility of the LFIA test strips for DFB detection in standard solutions (n = 6).
