# Correction: Zhu et al. A Sensitive, Rapid, On-Site Detection of Diflubenzuron in Food via a Colloidal Gold-Based Test Strip. *Foods* 2026, *15*, 977

**DOI:** 10.3390/foods15173100

**Published:** 2026-09-01

**Authors:** Yanni Zhu, Dan Wang, Wenqin Wu, Yinghua Deng, Zhaowei Zhang, Zhi-Quan Tian

**Affiliations:** 1 College of Chemistry and Molecular Sciences, Wuhan University, Wuhan 430072, China; 2023202030036@whu.edu.cn (Y.Z.); d202580180@hust.edu.cn (D.W.); 2 School of Bioengineering and Health, Hubei Hongshan Laboratory, Wuhan Textile University, Wuhan 430020, China; wenqin_wu@163.com; 3 State Key Laboratory of New Textile Materials and Advanced Processing Technologies, Wuhan Textile University, Wuhan 430020, China; 4 Hubei Key Laboratory of Purification and Application of Plant Anti-Cancer Active Ingredients, School of Chemistry and Life Science, Hubei University of Education, Wuhan 430205, China; dengyinghua@hue.edu.cn

**Error in Table**

In the original publication [1], there was a mistake in Table 2 as published. Several values were displayed after preliminary rounding during the unit-conversion step from working-solution concentrations (ng mL^−1^) to matrix-equivalent concentrations (µg kg^−1^). In addition, several very low blank-matrix responses below the reportable detection range were displayed numerically rather than as ND. The corrected Table 2 appears below.

**Text Correction**

There was an error in the original publication. The reported recovery and RSD ranges in the Abstract, Section 3.6, and the Conclusions should be updated to correspond to the corrected Table 2.

**A correction has been made to the Abstract:**

Validation in eight food matrices (milk, chicken, mushrooms, pear, Chinese cabbage, rice, dried chili, and peanut) showed recoveries of 97.0–110.2% with RSDs of 2.5–4.9%.

**A correction has been made to Section 3.6, Detection Performance of the Test Strip:**

To evaluate the performance of the developed test strips, recoveries and precision were assessed across eight food matrices. The results showed that recoveries ranged from 97.0% to 110.2%, with RSDs of 2.5–4.9%, demonstrating excellent accuracy and reproducibility.

Specifically, the spiking levels (expressed in µg kg^−1^ within the original matrices) and corresponding recoveries were as follows: milk, 98.3–102.6% (RSD 2.5–4.0%); chicken, 97.6–101.0% (RSD 3.8–4.5%); fresh mushrooms, 99.6–104.4% (RSD 2.7–3.9%); pear, 97.9–103.4% (RSD 3.3–4.9%); Chinese cabbage, 101.8–105.3% (RSD 2.9–4.0%); rice, 97.0–101.6% (RSD 2.7–4.4%); dried chili, 102.3–110.2% (RSD 3.1–4.4%); and peanut, 98.4–102.9% (RSD 3.9–4.7%) (Figure 6c).

**A correction has been made to the Conclusions:**

The test strips demonstrated good repeatability and inter-batch reproducibility (CV < 10%), satisfactory recovery rates (97.0–110.2%), and good storage stability for at least 60 days at 4 °C.

The authors state that the scientific conclusions are unaffected. This correction was approved by the Academic Editor. The original publication has also been updated.

## Figures and Tables

**Table 2:** Validation of the proposed LFIA method by comparison with LC-MS in actual food samples (*n* = 3).

		This Work			LC-MS		
Samples	Spiked (µg kg^−1^)	Detected (µg kg^−1^)	Recovery (%)	RSD (%)	Detected (µg kg^−1^)	Recovery (%)	RSD (%)
Milk	0.0	ND ^a^	—	—	ND ^a^	—	—
	50.0	51.3 ± 1.4	102.6	2.7	52.3 ± 1.6	104.6	3.1
	500.0	505.9 ± 20.1	101.2	4.0	508.2 ± 19.8	101.6	3.9
	1000.0	983.2 ± 24.3	98.3	2.5	984.8 ± 23.5	98.5	2.4
Chicken	0.0	ND ^a^	—	—	ND ^a^	—	—
	50.0	49.4 ± 1.9	98.8	3.8	50.4 ± 2.0	100.8	4.0
	500.0	504.8 ± 22.5	101.0	4.5	507.2 ± 21.8	101.4	4.3
	1000.0	976.1 ± 41.6	97.6	4.3	978.3 ± 40.2	97.8	4.1
Fresh mushroom	0.0	ND ^a^	—	—	ND ^a^	—	—
	50.0	52.2 ± 1.8	104.4	3.4	53.4 ± 1.9	106.8	3.6
	500.0	513.4 ± 20.2	102.7	3.9	514.9 ± 19.5	103.0	3.8
	1000.0	996.0 ± 26.5	99.6	2.7	998.2 ± 25.8	99.8	2.6
Pear	0.0	ND ^a^	—	—	ND ^a^	—	—
	50.0	51.3 ± 1.7	102.6	3.3	51.7 ± 1.8	103.4	3.5
	500.0	489.4 ± 23.6	97.9	4.8	491.1 ± 22.8	98.2	4.6
	1000.0	1033.6 ± 50.4	103.4	4.9	1036.3 ± 49.2	103.6	4.7
Chinese cabbage	0.0	ND ^a^	—	—	ND ^a^	—	—
	50.0	52.3 ± 1.5	104.6	2.9	52.9 ± 1.6	105.8	3.0
	500.0	508.9 ± 20.2	101.8	4.0	511.3 ± 19.6	102.3	3.8
	1000.0	1053.1 ± 40.9	105.3	3.9	1055.2 ± 39.8	105.5	3.8
Rice	0.0	ND ^a^	—	—	ND ^a^	—	—
	50.0	48.5 ± 1.3	97.0	2.7	49.8 ± 1.4	99.6	2.8
	500.0	508.2 ± 22.3	101.6	4.4	510.3 ± 21.5	102.1	4.2
	1000.0	987.4 ± 42.5	98.7	4.3	989.1 ± 41.2	98.9	4.2
Dried chili pepper	0.0	5.9 ± 0.2	—	3.4	5.9 ± 0.2	—	3.4
	50.0	55.1 ± 1.7	110.2	3.1	56.2 ± 1.8	112.4	3.2
	500.0	511.7 ± 22.6	102.3	4.4	514.4 ± 21.9	102.9	4.3
	1000.0	1047.5 ± 36.5	104.8	3.5	1050.2 ± 35.8	105.0	3.4
Peanut	0.0	ND ^a^	—	—	ND ^a^	—	—
	50.0	49.2 ± 2.2	98.4	4.5	50.3 ± 2.3	100.6	4.6
	500.0	508.6 ± 23.9	101.7	4.7	511.4 ± 23.2	102.3	4.5
	1000.0	1029.3 ± 40.5	102.9	3.9	1031.2 ± 39.8	103.1	3.9

^a^ ND: no reportable residue detected. Note: Low-level blank responses below the reportable detection range were reported as ND and not quantified. Working-solution concentrations were converted to matrix-equivalent concentrations using a factor of 10. Values slightly above 1000 µg kg^−1^ in spiked samples correspond to recoveries above 100% and were used only for recovery evaluation; unknowns above the linear range should be diluted and reanalyzed. Milk values assume a density of approximately 1 kg L^−1^.
