# Supplementary material for: A Sensitive, Rapid, On-Site Detection of Diflubenzuron in Food via a Colloidal Gold-Based Test Strip
Source: Foods. 2026 Mar 10;15(6):977. doi: 10.3390/foods15060977 (PMC13025240; doi:10.3390/foods15060977)
Supplement: Supplementary file 1 [file foods-15-00977-s001.zip › foods-4164257-supplementary.pdf]

Supplementary Material

A sensitive, rapid, on-site detection of diflubenzuron in food via a colloidal gold-based test strip

Yanni Zhu [1], Dan Wang [1], Wenqin Wu [2], Yinghua Deng [3], Zhaowei Zhang [2,*] and Zhi-Quan Tian [1,*]

1 College of Chemistry and Molecular Sciences, Wuhan University, Wuhan 430072, P.R. China

2 School of Bioengineering and Health, State Key Laboratory of New Textile Materials and Advanced Processing Technologies, Wuhan Textile University, Hubei Hongshan Laboratory, Wuhan, 430062, PR China

3 Hubei Key Laboratory of Purification and Application of Plant Anti-Cancer Active Ingredients, School of Chemistry and Life Science, Hubei University of Education, Wuhan, 430205, P.R. China

* Correspondence: zqtian@whu.edu.cn (Z.-Q.T.); zwzhang@whu.edu.cn (Z.Z.)

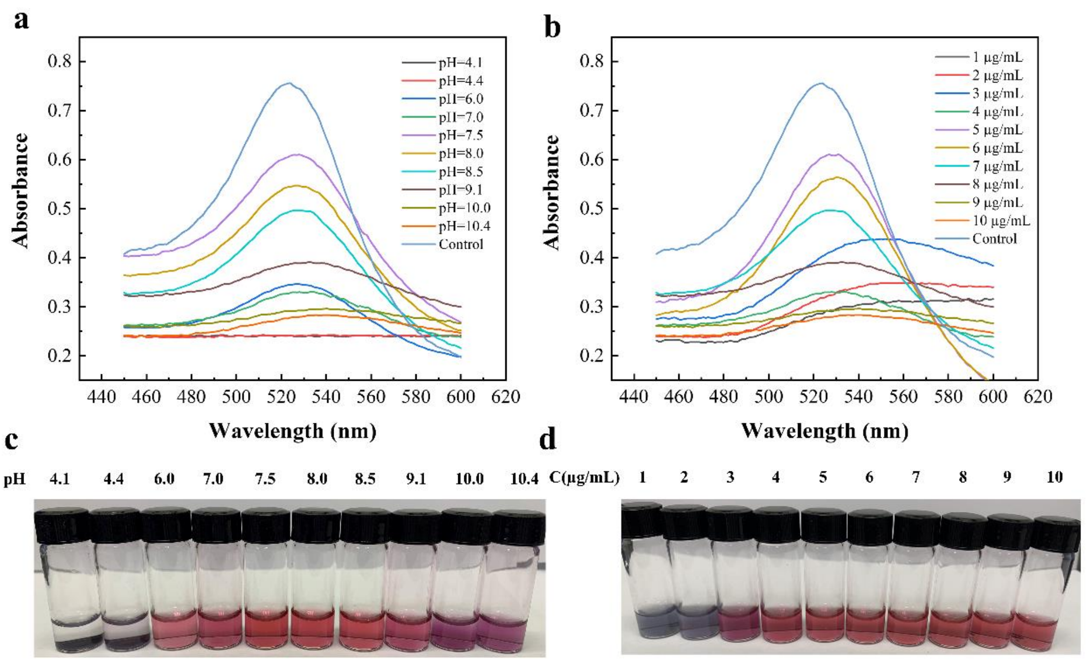

Figure S1. Optimization of pH and antibody concentration for colloidal gold immunoassay. (**a**) UV-Vis spectra of colloidal gold at different pH values; (**b**) UV-Vis spectra of colloidal gold with varying anti-diflubenzuron antibody concentrations; (**c**) Photographs of colloidal gold solutions at different pH values; (**d**) Photographs of colloidal gold solutions with varying anti-diflubenzuron antibody concentrations.

Table S1. Intra-day precision, inter-day precision, and batch-to-batch reproducibility of the LFIA test strips for DFB detection in standard solutions (n = 6).

Spiked concentration (μg kg⁻¹)	Intra-day			Inter-day			Between-batch		
	Detected ± SD (μg kg^{-1})	Recovery (%)	RSD (%)	Detected ± SD (μg kg^{-1})	Recovery (%)	RSD (%)	Detected ± SD (μg kg^{-1})	Recovery (%)	RSD (%)
0.1	0.086 ± 0.008	86.4	9.3	0.085 ± 0.008	85.1	9.8	0.092 ± 0.009	91.5	10.3
10	8.99 ± 0.75	89.9	8.4	9.12 ± 0.81	91.2	8.9	9.81 ± 0.65	98.1	6.6
100	94.5 ± 5.2	94.5	5.5	93.8 ± 6.1	93.8	6.5	96.2 ± 5.8	96.2	6.0

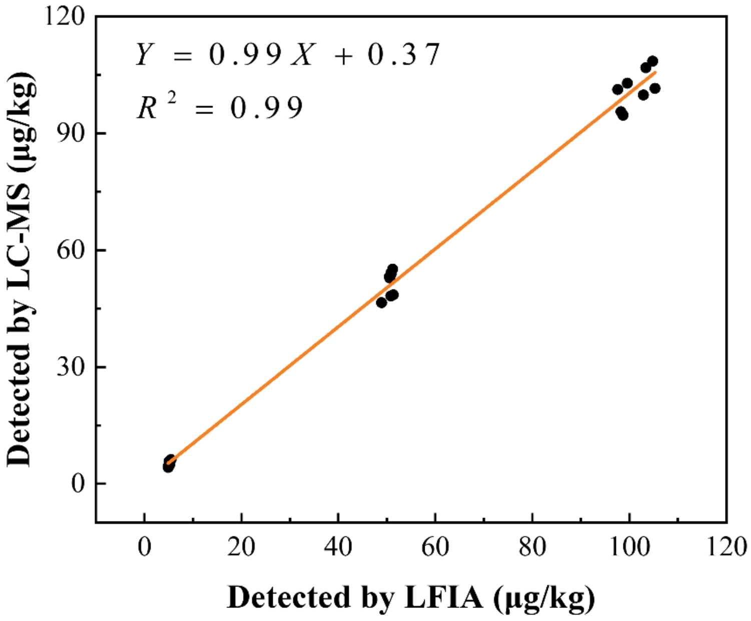

Figure S2. Correlation between the developed LFIA and the reference LC–MS method for DFB detection in spiked food samples.
